# Prevalence of helmet use among motorcycle users in Dar Es Salaam, Tanzania

**DOI:** 10.11604/pamj.2015.20.438.5659

**Published:** 2015-04-30

**Authors:** Cosmas George Kauky, Rogath Saika Kishimba, Loveness John Urio, Ahmed Mohammed Abade, Janneth Maridadi Mghamba

**Affiliations:** 1 Tanzania Field Epidemiology and Laboratory Training Programme (FELTP), Dar Es Salaam, Tanzania; 2 Ministry of Health and Social Welfare, Dar Es Salaam, Tanzania

**Keywords:** Prevalence, helmet, motorcycle, Tanzania

## Abstract

**Introduction:**

The purpose of this study was to determine prevalence of helmet use among motorcyclists as one of the preventive measures for road traffic injuries.

**Methods:**

A cross sectional observational survey was conducted in the 3 Districts (Kinondoni, Ilala and Temeke) that make Dar es Salaam. Tanzania. A standardized line-listing form and checklist were used to record the drivers and passengers use of helmet as observed by study investigators. Data for helmet use was collected on one weekday and one weekend day. Time for observation was during the rush hour in the morning, noon and evening. Then data were entered into Epi Info 3.5.1 analysis

**Results:**

A total of 7,678 motorcycle drivers and 4,328 passengers observed in this study. Drivers were almost male (98.8%) and 73.2% of all passengers were males. The prevalence use of helmet use among motorcyclist's riders was 82.1% and among passengers was 22.5%. Proportion of helmet use in drivers and passengers observed were relatively similar during weekday and weekend day and time of observation.

**Conclusion:**

This study showed the relative high helmet use among motorcyclist riders though very low in passengers. This study recommends increased community awareness on helmet use among passengers and enforcement and revival of road safety laws of passengers and motorcyclists on helmet use.

## Introduction

Road traffic injuries take an enormous toll on individuals and communities as well as on national economies. Middle-income countries, which are motorizing rapidly, are the hardest hit. Half of the world's road traffic deaths occur among motorcyclists (23%), pedestrians (22%) and cyclists (5%) [1]. Tanzania, a developing country in Africa, has witnessed at least a fivefold rise in recorded traffic-related fatalities during the last decade [2]. This in part is due to the proliferation of roads, which are often in poor states and also, a phenomenal increase in the number of motor vehicles, many of which are old, and not road-worthy. The increasing use of motorcycles particularly for commercial service is a source of concern in this regard because motorcycles cause many more fatal road crashes than other vehicles worldwide. As motorcycles are relatively unsafe vehicles, the riders must be considered as unprotected vehicle users and their injuries are usually severe [3]. Recently the prevalence of road traffic accidents in Tanzania had increased whereby motorcycle accidents form a large proportion and a fatal category of motor traffic accidents (Morbidity & mortality (16.7%) [4]. Non-use of helmet is a specific factor leading to head injuries and fatalities resulting from motorcycle crashes. In a study conducted by interviewing motorcycle riders at their parking points in Dar es Salaam 2011 revealed that (52.7%) were observed wearing helmet, although 91.8% of them had no passenger's helmet [2]. In northern region, Mwanza Motorcyclists accounted for the majority of motorcycle injury patients and Helmet use was found to be (22.7%) among the patient admitted with motorcycle injury [5]. Non-adherence of helmet use includes feelings of discomfort due to heat during the hot weather, and lateral vision and hearing ability impairment alcohol use and the altitude to the law implementation. However it has been shown that helmets do not impair hearing ability and the lateral vision can be complemented by lateral head rotation [4]. The government through the ministry of Home affairs department of police traffic and SUMATRA provide education on safety use of road, traffic regulations through different media including Radio, Television and magazine, and police penalty notification. Since a number of studies have been done with different prevalence, on which some reported to be low (22.7%) and some as high as 52.7% with different target population [6]. Most of these studies target causalities in the health facilities and others interviewing commercial motorcyclist with no observational studies reported on the general population. The purpose of this study was to determine prevalence of helmet use among motorcyclist as one of the safety measures in road traffic accidents which was targeted on the general population.

## Methods

**Study design:** A cross sectional survey was used whereby motorcyclist (commercial and non-commercial) and passengers were observed on helmet wearing practice at the main road car junctions which were randomly selected. Data were collected for the duration of two days (weekend and weekday) during the peak hours on road use.

**Study area:** The study was carried out in three municipals of Ilala, Kinondoni and Temeke in Dar es Salaam City. The specific sites were at the main road junctions with high traffic flow.

**Study population:** The study involved motorcycle riders and their passengers in Dar es Salaam where by the representative sample were observed at Morogoro Road (Ubungo junction), Sam-Nujoma Road (Mwenge junction), Alli Hassan Mwinyi Road (Sealender bridge junction), Uhuru road (TAZARA Junction), Chang'ombe road (Serengeti breweries junction) and Kilwa road (Temeke junction).

**Sample size:** Sample size was determined by the availability of motorcycle riders with or without passengers at the observation points within the time observation which was two hours per period. Assume 52% of helmet users in Dar es salaam (Mwakapasa E.G 2011 - 6), then alpha = 0.05(i.e. 95% CI), precision of 3% and adjusting for design effect = 3 for selecting specific junctions out of in Dar, adjusting for double counting of 0.25 (i.e. 1:4 cyclist might be seen by multiple observers at different junctions at same observation window or same individual seen at different at different time window but at the same spot). This gives a minimum sample of**3974**.

**Sampling technique:** Study sites were selected considering high traffic flow, at study site the residents began observations at the traffic lights, by selecting the second line of motorcycles from traffic lights in a stop position and observe consecutive motorcycles until the vehicles start moving (one traffic light cycle). The residents then walked back to the traffic light and start the process again when the motorcycles are in the stop position

**Data collection:** Study investigators worked in pairs observing at different route to collect data at the assigned study sites in the six junctions with high traffic flow.

**Period for data collection**: Data were collected in three sessions during the weekday and two sessions during the weekend day. The sessions on the weekday were during the rush hour in the morning from 7 .00- 9.00 AM, 12.00- 2.00 PM and 5.00-6.00 PM. The weekend sessions were from 9.30 PM to 11.30 PM and 5.00 -6.00PM.

**Data collection tool:** Standardized observation checklist was used to record the driver‘s and passengers’ sex, use of helmet and number of passengers per motorcycle.

**Data analysis and management:** Data from standardized observation checklist were individually coded and entered into Epi Info 3.5.1; individual data were merged and later analyzed. Descriptive statistics and tables were generated from the program to calculate helmet prevalence in Dar es Salaam.

**Ethical Considerations**: The study obtained ethical clearance from MUHAS high degree ethical committee of research and publication. Permission to do the study was obtained from Surface and Marine Transport Regulatory Authority (SUMATRA)

## Results

**Figure 1:**
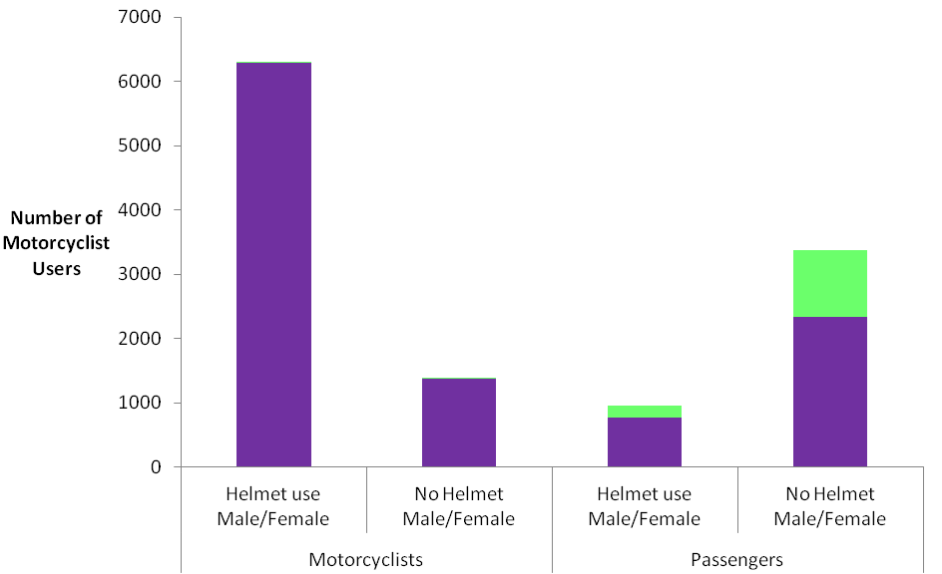
Distribution of motorcyclists and passengers by sex and helmet use status, Dar es Salaam, December, 2013

**Figure 2:**
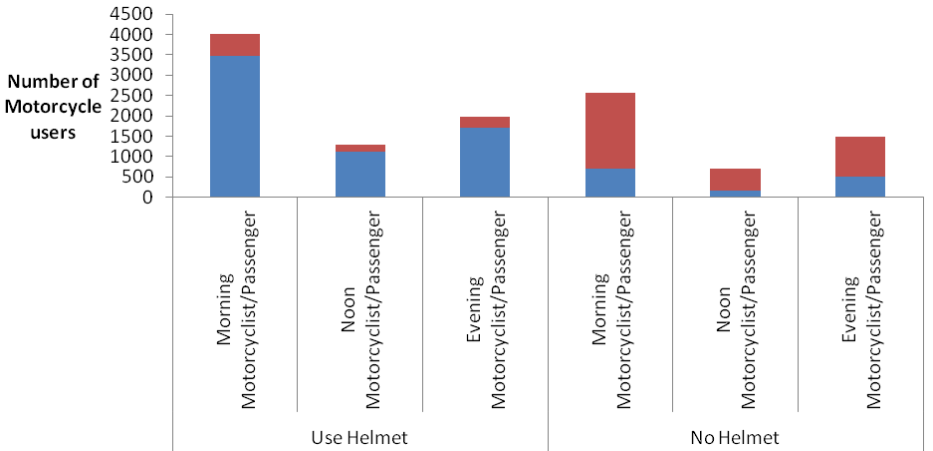
Distribution of motorcyclists and passengers by time of observation and helmet use status, Dar es salaam, December, 2013

**Figure 3:**
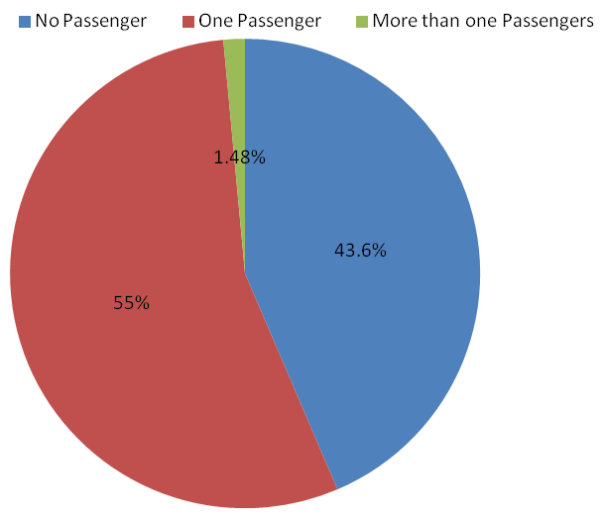
Distribution of motorcyclists by number of passengers, Dar es salaam, December, 2013

A total of 7678 motorcyclists were observed with male preponderance 7660 (99.8%). More than half 4334 (56.4%) of motorcyclists at least had a passenger of whom majority 3173 (73.2%) were males. Table 1 shows the distribution of motorcyclists by sex at different observational sites. All observational sites had almost proportionate motorcyclists except Sea-ender junction and TAZARA junction which had 14.1% and 12.9% respectively. The distribution of motorcyclists and passengers by helmet use status; majority 6,302 (82.1%) of motorcyclist wore helmet while very few 965 (22.5%) passengers were observed to use helmet. And therefore the prevalence of helmet use among motorcyclists was estimated at 82.1%. Figure 1 shows the distribution of motorcyclists and passengers by sex and helmet use status. Majority 3369 (77.7%) of passengers did not use helmet. Most 2340 (69.5%) of passengers who did not wear helmet were males. Helmet use behaviour among males and females motorcyclists were not apparent as there were very few 18 (0.2%) female motorcyclists that were observed. The distribution of motorcyclists and passengers by day of observation and helmet use status showed high proportion 2040 (76.4%) and 1329 (79.8%) of passengers were observed not to use helmet in a weekday and weekend day respectively. Figure 2 shows the distribution of motorcyclists and passengers by time of observation and helmet use status. Majority 3464 (83%), 1123 (87.2%), 1715 (77.4%) of motorcyclists wore helmet during all time of observations; morning, noon and evening respectively. There was a proportionate high 1863 (77.5%), 527 (75.5%), 979 (79.5%) proportion of passengers who did not wear helmet during all times of obervation; morning, noon and evening respectively. Figure 3 shows distribution of motorcyclists by number of passengers. Majority 4218 (55%) of motorcyclists have only one passenger in their motorbike.

**Table 1:** Distribution of motorcyclists by sex at different observational sites, Dar es Salaam, December, 2013

	Male n (%)N = 7660	Female n (%) N = 18	Total n (%)N = 7678
Mwenge	1494 (19.5)	1 (5.6)	1495 (19.5)
Kilwa	1431 (18.7)	3 (16.7)	1434 (18.7)
Ubungo	1362 (17.8)	6 (33.3)	1368 (17.8)
Chang'ombe	1300 (17.0)	7 (38.9)	1307 (17.0)
Sealender	1079 (14.1)	1 (5.6)	1080 (14.1)
TAZARA	994 (13.0)	0 (0.0)	994 (12.9)
Total	7660 (100)	18 (100)	7678 (100)

## Discussion

The purpose of the study was to determine the prevalence of helmet use among motorcyclist in Dar es Salaam, The results of this study showed the relative high proportion of helmet use among motorcyclist riders (82.1%)) and low in passengers (22.5%) in Dar es salaam, Tanzania. This indicates that the large proportion of passengers are not using motorcycle helmet. The rider helmet use proportion of 82.1% and passenger (22.5%) is higher compared to previous study done 2011 which showed 52.7% and 8.2% respectively [6]. Our study showed a higher proportion of helmet use as compared to rate obtained in studies conducted in Vietnam (34.7%) and Ghana (34.2%) [7, 8]. However we found a relatively similar rate with Indonesia (89%) and China (63%) [9, 10]. Again the rate of helmet use among passengers in our study were relatively similar to passenger's rates found in Vietnam (18.9%), Indonesia (20%), and China (29%) [7, 9, 10]. However our rate of helmet use among passengers were much higher more than ten folds of what was found in Ghana (1.9%) [8]. The higher helmet use rate among as compared to passengers in this study is in accordance with other studies [7–9]. The low proportion of helmet use among passengers may be explained by the fact that in Tanzania most passengers and raiders are not aware that they are supposed to wear helmet or not well informed of legislation on the helmet usage. The traffic police behavior on helmet use enforcement among riders alone may also explain our low helmet use rate among passengers. In a study done in Ghana awareness on helmet use among both raiders and passengers as well as helmet use law enforcement among raiders only were major factors contributed to low helmet use among passengers [8]. This study found relatively similar proportion in helmet use in weekdays and weekends and the time of observation and Helmet use behavior among males and females motorcyclists were not apparent as there were very few Study limitation observed was double counting, however, observation was only done at the direction of high flow of traffics to minimize double counting.

## Conclusion

The results of the study showed the jprevalence of helmet use by motorcyclists observed in Dar Es Salaam, relatively high in motorcycle riders though very low in passengers. This indicates there are safety benefits of helmet to increase the prevalence of helmet use among passengers. Continuously education on the importance of helmet use to the community should be addressed through Medias and special campaign. The sustained enforcement of the road traffic law by traffic police to help increase helmet use among motorcycle users should be continuously done in Tanzania. The government and Nongovernmental organization dealing with road traffic prevention should ensure that standard helmets are made available and at affordable price to both motorcyclist and passenger.
